# Bactericidal activity of three different antiseptic ophthalmic preparations as surgical prophylaxis

**DOI:** 10.1007/s00417-021-05361-3

**Published:** 2021-08-24

**Authors:** Daniele Tognetto, Marco R. Pastore, Gian Marco Guerin, Giuliana Decorti, Martina Franzin, Cristina Lagatolla, Gabriella Cirigliano

**Affiliations:** 1 Department of Medical, Surgical Sciences and Health, Eye Clinic, University of Trieste, Piazza dell’Ospitale 1, 34129 Trieste, Italy; 2 Institute for Maternal, Child Health IRCCS Burlo Garofolo, Trieste, Italy; 3 Department of Medical, Surgical Sciences and Health, University of Trieste, Trieste, Italy; 4 Science of Reproduction and Development, University of Trieste, Trieste, Italy; 5 Department of Life Sciences, University of Trieste, Trieste, Italy

**Keywords:** Antiseptic, Microbicidal, Iodine, Ophthalmology, Ozone, Chlorhexidine, Infection

## Abstract

**Purpose:**

In the era of antibiotic resistance, there is an increased interest in antiseptic solutions that might represent a reliable option for ocular surface disinfection. The objective of this study is to compare for the first time three different antiseptic ophthalmic preparations to assess their in vitro antimicrobial activity.

**Methods:**

The antiseptic activity of three commercial ophthalmic solutions, IODIM (povidone-iodine 0.6% in hyaluronic acid vehicle—Medivis, Catania, Italy), OZODROP (nanoemulsion with ozonated oil—concentration not specified—FBVision, Ophthalmic Pharmaceuticals, Rome, Italy), and DROPSEPT (chlorhexidine 0.02% and vitamin E 0.5% Tocopherol Polyethylene Glycol 1000 Succinate—TPGS, Sooft Italia, Montegiorgio, Italy), was tested in vitro on six reference strains by time-killing assays. Viable cells were evaluated after 1, 15, 30 min; 2, 6, and 24 h exposure by seeding 100 µl of the suspension (or appropriate dilutions) on LB agar or Sabouraud-dextrose agar. All plates were incubated at 37 °C for 24 h and evaluated by manually counting the colonies.

**Results:**

IODIM solution showed a very rapid microbicidal activity: the number of viable cells for all the tested strains was under the detection limit (less than 10 CFU/ml) already after 1 min exposure, and this result was maintained at every incubation time. The rapid antimicrobial activity of povidone-iodine was not replicated when testing the other two antiseptics.

**Conclusions:**

The study reports the great efficacy in reducing bacterial load in a very short time of povidone-iodine 0.6% compared with other antiseptic preparations.


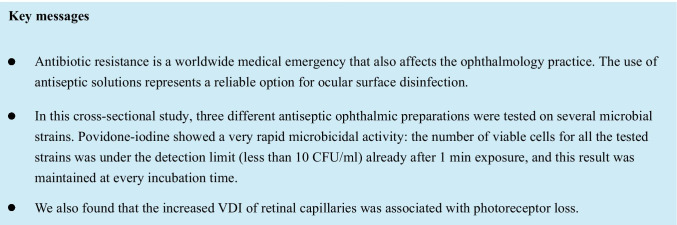


## Introduction

Nowadays, antibiotic resistance is a worldwide medical emergency that also affects the ophthalmology practice [1]. New resistance mechanisms are emerging and spreading globally, threatening our ability to treat common infectious diseases [2]. The antibiotic resistance crisis has been attributed to their overuse and misuse, and to the lack of new drug development by the pharmaceutical industry due to reduced economic incentives and challenging regulatory requirements [3].

Indeed, it is mandatory to achieve adequate antisepsis of the ocular surface in the pre-operative setting using both topical antibiotics and disinfectant ophthalmic solutions [4, 5].

Povidone-iodine (PVP-I) is an iodinated polyvinyl polymer used for years as a disinfectant and antiseptic agent, especially in pre-operative preparation of the skin and mucous membranes, as well as for the treatment of contaminated wounds [6]. The iodine molecules are free to oxidize vital pathogen structures such as amino acids, nucleic acids, and membrane components [7].

Currently, regimens for prophylaxis against postsurgical endophthalmitis include the use of PVP-I except in case of allergy [8–10]. Povidone-iodine is used worldwide due to its wide-spectrum antimicrobial activity, absence of resistant bacteria, and low cost. In a recent study, Musumeci et al. have demonstrated that PVP-I 0.6% has a more rapid bactericidal activity than PVP-I 5%, due to the greater amount of free iodine when PVP-I is in low concentration [11].

Ozone is the most powerful oxidizing agent found in nature yet known in medicine for its antiseptic and anti-inflammatory properties [12]. The introduction of oxidizing agents in drops for disinfection of the ocular surface is very recent. Their use has been advocated on the basis of efficacy against all microorganisms, as well as the lack of induction of antibiotic resistance [13]. The ozonated oil generates reactive oxygen species and lipid peroxidation products, which cause disruption of membrane layers, oxidation of nucleosides and aminoacids, and, ultimately, cell death [14]. To make it tolerated by the ocular surface, a nanoemulsion with ozonated oil within liposomes in solution with hypromellose and deionized water has been recently placed on the market (OZODROP, FBVision, Ophthalmic Pharmaceuticals, Rome, Italy) [15].

Chlorhexidine is an alternative antiseptic that was first employed in ophthalmology as a disinfectant for soft contact lenses and has been used to treat *Acanthamoeba* keratitis for more than 20 years [16, 17]. It is a cationic biguanide that binds to and disrupts the bacterial cell wall leading to cytoplasmic damage and cell death. Chlorhexidine has been demonstrated to be effective when investigating ocular bacterial count after antisepsis [16, 18]. In a multicentre retrospective case series using aqueous chlorhexidine, the endophthalmitis rate was 0.0074%, which is comparable with povidone-iodine rates [19]. Although chlorhexidine compares well with povidone-iodine preparation, especially for patients intolerant of the latter, it is not sporicidal, and there are reports of reduced susceptibility in methicillin-resistant *Staphylococcus aureus* and fungi [20–22]. A new preparation based on chlorhexidine was recently introduced on the market DROPSEPT (Sooft Italia, Montegiorgio, Italy). It is a 0.5% solution which includes vitamin E and Tocopherol Polyethylene Glycol 1000 Succinate.

The purpose of this study is to compare for the first time three different antiseptic ophthalmic preparations in order to assess their in vitro antimicrobial activity.

## Materials and methods

In this experimental study, the antiseptic activity of three commercial ophthalmic solutions, IODIM (povidone-iodine 0.6% in hyaluronic acid vehicle), OZODROP (nanoemulsion with ozonated oil—concentration not specified) [15], and DROPSEPT (chlorhexidine 0.02% and vitamin E TPGS 0.5%) [23], was tested in vitro on the following reference strains: *Staphylococcus aureus* ATCC 25923 (methicillin-susceptible—met^S^), *Staphylococcus aureus* ATCC 43,300 (methicillin-resistant—met^R^), *Staphylococcus epidermidis* ATCC 12228, *Pseudomonas aeruginosa* ATCC 27853, *Escherichia coli* ATCC 25922, and *Candida albicans* ATCC 90028. Microbial strains were incubated overnight at 37 °C in LB broth (bacterial strains) or in Sabouraud-dextrose broth (*Candida*) and the optical density of the cultures was measured at 600 nm. Based on growth curves previously obtained for each strain, microbial suspensions in phosphate buffered saline solution at a concentration of about 5 × 10^8^ colony forming units (CFU)/ml were prepared. Ten microliters of each suspension was added to 1 ml of each ophthalmic solution to achieve a final concentration of about 5 × 10^6^ CFU/ml. For the determination of the microbicidal activity, viable cells were evaluated at 1, 15, 30 min; 2, 6, and 24 h by seeding 100 µl of the suspension (or proper dilutions when needed) on LB agar or Sabouraud-dextrose agar. All plates were incubated at 37 °C for 24 h and evaluated by manually counting the colonies. All experiments were performed in triplicate and positive and negative controls were included in each experiment. Results were expressed as the mean value of the log_10_ CFU/ml ± standard deviation.

## Results

The antiseptic activity of the three ophthalmic solutions against the tested strains was evaluated at different exposure times.

IODIM solution showed a very rapid microbicidal activity: the number of viable cells for all the tested strains was under the detection limit (less than 10 CFU/ml) already after 1 min exposure, and this result was maintained at every incubation time (Table 1).

**Table 1:** Microbial growth at different times after exposure to povidone-iodine 0.6% (IODIM)

	0	1’	15’	30’	2 h	6 h	24 h
*S. epidermidis* ATCC 12228	6.25 ± 0.19	No growth	No growth	No growth	No growth	No growth	No growth
*S. aureus* met^S^ ATCC 25923	6.56 ± 0.03	No growth	No growth	No growth	No growth	No growth	No growth
*S. aureus* met^R^ ATCC 43300	6.62 ± 0.20	No growth	No growth	No growth	No growth	No growth	No growth
*E. coli* ATCC 25922	6.30 ± 0.08	No growth	No growth	No growth	No growth	No growth	No growth
*P. aeruginosa* ATCC 27853	6.43 ± 0.10	No growth	No growth	No growth	No growth	No growth	No growth
*C. albicans* ATCC 90028	6.21 ± 0.57	No growth	No growth	No growth	No growth	No growth	No growth

*met*^*S*^ methicillin-susceptible; *met*^*R*^ methicillin-resistant

The rapid antimicrobial activity of PVP-I was not replicated when testing the other two antiseptics. As summarized in Table 2, liposome-vehiculated ozonated oil (OZODROP) showed a weak killing activity only after prolonged exposure against three out of the six tested strains. Indeed, after 24-h exposure, it reached, as best result, a reduction of about 2 log_10_ of the CFUs for *S. epidermidis* and of 1 and 0.5 log_10_ for *E. coli* and *P. aeruginosa*, respectively. No activity was detected against the two *S. aureus* strains and *C. albicans*.

**Table 2:** Microbial growth at different times after exposure to nanoemulsion with ozonated oil (OZODROP)

	0	1’	15’	30’	2 h	6 h	24 h
*S. epidermidis* ATCC 12228	6.31 ± 0.26	6.25 ± 0.19	6.13 ± 0.24	6.31 ± 0.35	6.25 ± 0.10	6.25 ± 0.19	4.63 ± 0.45
*S. aureus* met^S^ ATCC 25923	6.56 ± 0.03	6.77 ± 0.27	6.81 ± 0.26	6.72 ± 0.23	6.72 ± 0.09	6.67 ± 0.07	6.57 ± 0.23
*S. aureus* met^R^ ATCC 43300	6.62 ± 0.20	6.85 ± 0.06	6.67 ± 0.05	6.67 ± 0.06	6.87 ± 0.12	6.78 ± 0.07	6.55 ± 0.27
*E. coli* ATCC 25922	6.57 ± 0.30	6.46 ± 0.48	6.43 ± 0.27	6.56 ± 0.25	6.57 ± 0.22	6.15 ± 0.41	5.41 ± 0.19
*P. aeruginosa* ATCC 27853	6.29 ± 0.54	6.43 ± 0.60	6.46 ± 0.57	6.47 ± 0.41	6.62 ± 0.11	6.10 ± 0.54	5.66 ± 0.19
*C. albicans* ATCC 90028	6.33 ± 0.21	6.30 ± 0.06	6.29 ± 0.02	6.37 ± 0.07	6.33 ± 0.18	6.32 ± 0.00	6.27 ± 0.13

*met*^*S*^ methicillin-susceptible; *met*^*R*^ methicillin-resistant

Regarding the chlorhexidine-containing ophthalmic solution (DROPSEPT), a good antiseptic activity against *E. coli* was detected from 2-h exposure onwards, reaching a complete killing after 24 h (Table 3). A weaker activity was observed against the three staphylococci strains (1–2 log_10_ CFUs reduction at 24 h), while *C. albicans* was not affected at all. For *P. aeruginosa*, the treatment showed a weak bactericidal activity after 2- and 6-h exposure, causing a reduction of the CFUs of about 1.5 log_10_ followed, at 24 h, by a partial regrowth of the microorganism. However, this is not really unexpected, as *Pseudomonas*’ ability to survive and even grow in different antiseptic solutions, including chlorhexidine, has been documented for a long time [24].

**Table 3:** Microbial growth at different times after exposure to chlorhexidine 0.02% (DROPSEPT)

	0	1’	15’	30’	2 h	6 h	24 h
*S. epidermidis* ATCC 12228	6.15 ± 0.10	6.15 ± 0.12	6.09 ± 0.12	5.94 ± 0.31	5.85 ± 0.20	5.66 ± 0.12	4.38 ± 0.48
*S. aureus* met^S^ ATCC 25923	6.46 ± 0.20	6.79 ± 0.16	6.76 ± 0.12	6.61 ± 0.20	6.34 ± 0.08	6.37 ± 0.10	5.72 ± 0.25
*S. aureus* met^R^ ATCC 43300	6.61 ± 0.20	6.63 ± 0.09	6.57 ± 0.10	6.56 ± 0.19	6.48 ± 0.31	5.93 ± 0.16	4.40 ± 0.13
*E. coli* ATCC 25922	6.46 ± 0.35	6.40 ± 0.46	6.09 ± 0.26	6.01 ± 0.22	5.81 ± 0.78	5.11 ± 0.30	No growth
*P. aeruginosa* ATCC 27853	6.82 ± 0.18	6.57 ± 0.41	6.74 ± 0.21	6.42 ± 0.26	5.33 ± 0.02	5.22 ± 0.07	7.75 ± 0.13
*C. albicans* ATCC 90028	6.33 ± 0.18	6.49 ± 0.09	6.50 ± 0.12	6.27 ± 0.10	6.42 ± 0.17	6.31 ± 0.08	6.34 ± 0.15

*met*^*S*^ methicillin-susceptible; *met*^*R*^ methicillin-resistant

## Discussion

With recent reports of emerging resistance to antibiotics, including ampicillin and vancomycin, attention has turned to the use of broad-spectrum antiseptics. In this study, the antimicrobial activity of three different antiseptic ophthalmic preparations was compared for the first time. The microbiological results clearly show the antimicrobial efficacy of povidone-iodine 0.6% after a short exposure time against all bacterial strains and fungi taken into examination in this experiment.

Povidone-iodine solutions in ocular site disinfection have already been studied on large-scale projects assessing its effectiveness as an antiseptic as well as its safety profile [25].

Compared to most antibiotics, a broad-spectrum antiseptic reduces the likelihood of resistance due to multiple mechanisms of action targeting different characteristics of cell biology [7, 19]. Indeed, despite its long history of efficacious use, no significant cases of microbial resistance to iodine have emerged. In contrast to PVP-I, bacterial resistance to chlorhexidine, quaternary ammonium salts, silver, and triclosan has been documented [7]. Furthermore, numerous studies have shown that PVP-I has a wider antimicrobial spectrum than other available antiseptics [26, 27].

The antiseptic and antiviral activity of ozonized oil in liposomes are well known, and its safety has been assessed in vitro and in vivo [28]. Looking at our results, we might guess that the antimicrobial activity lag was due to an inadequate concentration of the ophthalmic solution that did not reach the MIC and to the presence of liposomes that allow a gradual effect. Indeed, in clinical experience, the administration of OZODROP consists of one eye drop four times a day.

Looking at the number of CFUs after chlorhexidine exposure, it has been suggested that the onset of action of chlorhexidine is less immediate compared with PVP-I [29]. Chlorhexidine is widely used in antiseptic products, at a concentration that ranges from 0.12 in oral rinses to 4% for hand disinfection [29, 30]. The low efficacy in vitro observed in this study might be related to the lower concentration of the antiseptic (0.02%). However, it is necessary to point out that the formulation with the vitamin E TPGS was conceived to provide an encapsulation of chlorhexidine that should improve its absorption towards the corneal stroma but, on the other side, might delay its activity in vitro. In the light of these differences, it is mandatory to understand how the microbial flora on the ocular surface respond in vivo to clarify if the tear film, with its composition and its antibacterial enzymes and antibodies, might induce a different efficacy of the antiseptic molecules [31].

The results would indicate that we should use higher concentration or increase the frequency of the drops instillation to reach an adequate inhibitory concentration.

Our study results emphasize the great efficacy in reducing bacterial load in a very short time of povidone-iodine compared with other antiseptic preparations.

According to these results, the range of indications for topical use of antibiotics might decrease, with PVP-I as the main perioperative antiseptic measure. PVP-I has a rapid antiseptic activity, is readily available worldwide, its use is economically reasonable, and it does not induce microbial resistance. Therefore, PVP-I should outpace the prophylactic antibiotic before any ophthalmic surgical procedure, avoiding the onset of new antibiotic resistance. In patients with iodine allergy, the use of different antiseptic ophthalmic preparations must be taken into account.

Additional studies are required to assess the optimal timing, concentration, and exposure time within different ophthalmic procedures.
